# Age at childbirth and change in BMI across the life-course: evidence from the INCAP Longitudinal Study

**DOI:** 10.1186/s12884-022-04485-6

**Published:** 2022-02-24

**Authors:** Mónica Mazariegos, Jithin Sam Varghese, Maria F Kroker-Lobos, Ann M DiGirolamo, Manuel Ramirez-Zea, Usha Ramakrishnan, Aryeh D Stein

**Affiliations:** 1 INCAP Research Center for the Prevention of Chronic Diseases (CIIPEC), Institute of Nutrition of Central America and Panama (INCAP), 6 Avenida 6-25 zona 11, Guatemala City, Guatemala; 2 Nutrition and Health Sciences Doctoral Program, Laney Graduate School, Rollins School of Public Health, Emory University, 1518 Clifton Rd NE #7007, GA 30322 Atlanta, USA; 3 Georgia Health Policy Center, Georgia State University, 33 Gilmer Street SE, GA 30303 Atlanta, USA; 4 Hubert Department of Global Health, Rollins School of Public Health, Emory University, 1518 Clifton Rd NE #7007, GA 30322 Atlanta, USA

**Keywords:** Parity, Childbirth, Obesity, BMI, Timing, Reproductive age

## Abstract

**Background:**

Parity has been associated with both short- and long-term weight gain in women. However, it is not clear if timing of parity across the reproductive age has different associations with BMI.

**Methods:**

To prospectively assess the association between age at childbirth and maternal change in BMI, we analyzed data from the ongoing INCAP Longitudinal Study, which started in 1969 in four villages in Guatemala. Cohort women (n=778) provided information on reproductive history and anthropometric measures were measured in 1988-89 (adolescence, 15 to 25y), 2002-04 (early adulthood, 26 to 36y) and 2015-17 (mid adulthood, 37 to 55y). We evaluated the associations of number of live births in the period preceding each study wave (1969-77 to 1988-89, 1988-89 to 2002-04 and 2002-04 to 2015-17) with BMI change in the same period using multivariable linear regression models.

**Results:**

Number of live births between 1988 and 89 and 2002-04 was positively associated with increased BMI, while there was not an association between number of live births and BMI in the other intervals. Women who had one, two, or three or more children between 1988 and 89 and 2002-04 had 0.90 (kg/m^2^, 95% CI: -0.55, 2.35), 2.39 (kg/m^2^, 95% CI: 1.09, 3.70) and 2.54 (kg/m^2^, 95% CI: 1.26, 3.82) higher BMI, respectively, than women who did not give birth in the same period.

**Conclusions:**

Our findings suggest that women who had three or more children during early adulthood gained more weight compared to women who had no children in the same period. In contrast, women who had children earlier or later in their reproductive lives did not gain additional weight compared to those who did not have children during that period. Childbirth may have different associations with BMI based on the mother’s age.

**Supplementary Information:**

The online version contains supplementary material available at 10.1186/s12884-022-04485-6.

## Introduction

Maternal obesity has detrimental consequences to the health and wellbeing of both mother and child [1]. Parity and parity-related factors such as excessive gestational weight gain and postpartum weight retention have been associated with both short and long-term weight gain in women [1–4]. However, it is not clear whether pregnancies exert a cumulative weight gain with increasing parity or whether pregnancies that occur at particular ages are differentially related to weight gain. Previous research has not assessed the timing of parity, ignoring if timing of childbirth may explain the increased risk of weight gain and adiposity.

**Fig. 1:**
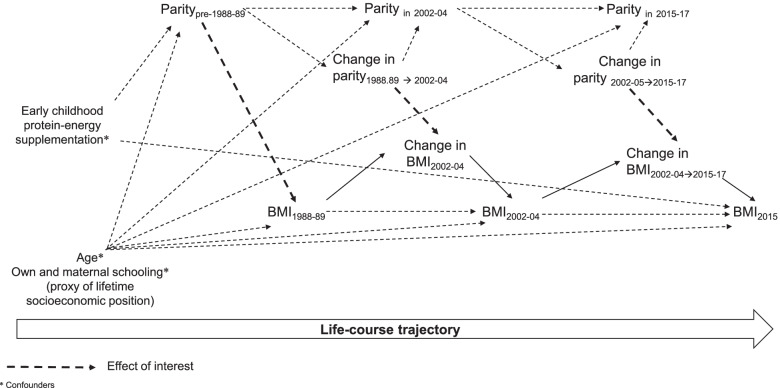
Conceptual framework for the association between timing of parity and maternal change in BMI along the life-course

Human reproductive life span (from menarche to menopause) can last more than thirty years [5, 6], and women can have children at any time during this period. Thus, parity can occur in a changing individual due to time-varying characteristics (e.g. women may begin each pregnancy at a different weight that the previous pregnancy) and in a changing environment (e.g. epidemiological and nutrition transitions). Life-course epidemiology suggests that the effect of an exposure on a health outcome may be dependent on the duration or timing of exposure [7]. Therefore, a careful understanding of childbirth during certain life periods taking an approach that considers factors across the life-course that influence the timing of reproductive events (e.g., onset of menarche, fertility, menopause), and considering the temporal ordering of exposure variables and the potential interaction with changing contexts over time, and recognizing the influence of reproductive health on chronic disease risk later in life is needed [8]. There are biological and social factors that may influence the association between timing of parity and maternal change in measures of obesity, including Body Mass Index (BMI) and waist circumference, across the life-course (See Fig. 1). Therefore, studies evaluating the timing of parity on different measures of adiposity can provide a better understanding of women’s weight gain patterns across the life-course and provide information on the most appropriate time across the life-course (windows of opportunity) for prevention and management of maternal obesity. A high BMI has been associated with morbidity and all-cause of mortality [9], while waist circumference provides both independent and additive information to BMI for predicting morbidity and risk of death [10]. Long-term changes in BMI and waist circumference in reproductive years may provide information beyond current weight for chronic disease risk [11]. Therefore, the aim of this study was to prospectively assess the association between timing of parity and maternal change in BMI in a well-established Guatemalan cohort.

## Methodology

### Study population

We analyzed data from the INCAP Longitudinal Study from Guatemala [12]. This cohort started as a community-randomized cohort trial designed to assess the effects of protein-energy nutritional supplement (*Atole*) on human development [13]. Protein deficiency was identified as the main cause of malnutrition at the time the study was planned, so the focus was on improving protein malnutrition while assuring enough extra energy to allow for protein use; the supplement was designed to be additive to the children’s diet. Four communities were randomly assigned in pairs to *Atole* – an energy and protein drink made from dry skimmed milk, sugar, and Incaparina (a vegetable protein mixture developed by INCAP [6.4 g protein per 100 mL, 0.4 g fat per 100 mL, 90 kcal per 100 mL]) or *Fresco* – a low-energy drink (all calories from sugar, 33 kcal/100 mL). Both drinks were similarly fortified with micronutrients in equal quantities per unit volume [13]. Children were included if they were < 7 y at the time of study launch or were born during the supplementation period, with follow-up until age 7 y or until the study ended in 1977. Full details of the original trial are published elsewhere [13].

Since the end of the original supplementation trial in 1977, the study population has been followed prospectively [14]. Data on sociodemographic, lifestyle, reproductive history, medical history, and anthropometric measurements have been collected.

Data for this analysis were collected during the original study (1969-77) and during follow-up surveys that were conducted in 1988-89 (at participant age 15 to 25y, which we consider adolescence), 2002-04 (at participant ages 26 to 36y, considered to be early adulthood) and 2015-17 (participants ages 37 to 55y, mid-adulthood).

**Fig. 2:**
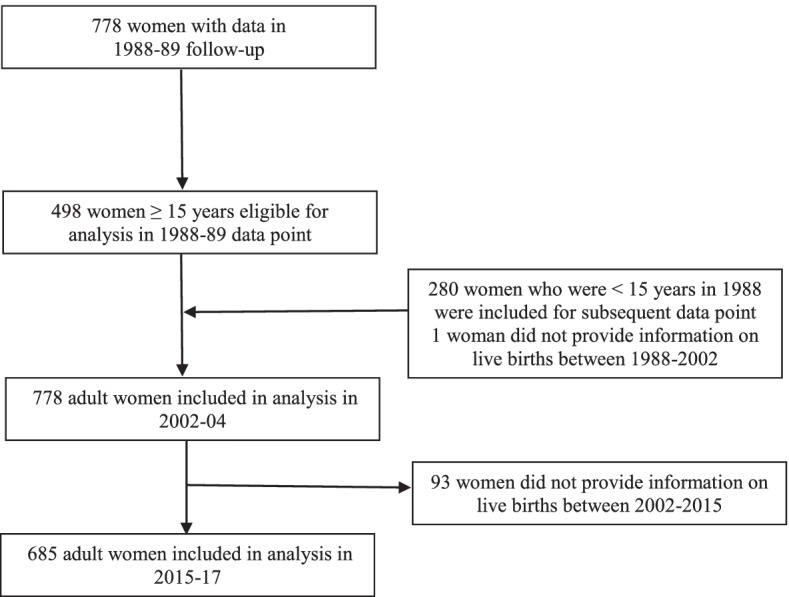
Flowchart of sample selection process

 Our analytical sample initially included all women who were ≥ 15 years (n=498) and participated in the 1988-89 follow-up. We then incorporated 280 additional women who were under 15 years in 1988-89 but reached adulthood by 2002-04, resulting in 778 women for analysis at that time point. Finally, we excluded 93 women who did not provide information on live births between 2002 and 04 and 2015-17, ending with a sample of 685 women for the 2015-17 time point (Fig. 2).

 All data collection followed protocols that were approved by the Institutional Review Boards of Emory University (Atlanta, GA) and INCAP (Guatemala City, Guatemala). All participants gave written informed consent at each survey wave.

### Assessment of parity

Parity was defined as the total number of live births, as determined by maternal recall at the 1988-89, 2002-04 and 2015-17 follow-ups. We categorized the number of live births within each interval (up to 1988-89; 1988-89 to 2002-04; 2002-04 to 2015-17) as: 0, 1, 2, 3, and ≥4 children, except for the period up to 1988-89, in which no women had 4 or more children. Information on miscarriages and stillbirths was not collected.

We evaluated data quality by checking that live births reported on the current follow-up were equal or greater than live births reported in the previous one. The 8 (1%) women with discrepant live birth histories were excluded from analysis.

### Assessment of BMI and change in BMI over time

Maternal weight (kg) and height (m) were measured in duplicate using standard techniques at each follow-up wave by trained and standardized personnel. Weight was measured using a calibrated scale with a precision of 100 g and height with a portable stadiometer with a precision of 0.5 cm. BMI (kg/m^2^) was calculated as weight (kg) divided by height (m) squared at each follow-up wave and we modeled it as a continuous variable. We calculated the change in BMI between 1988 and 89 and 2002-04 and between 2002 and 04 and 2015-17. In addition, we calculated the total BMI change between 1988 and 89 and 2015-17.

### Covariates

Data on socioeconomic factors were collected by interview at each follow-up wave. Potential covariates were identified a priori and included age (in years), own and maternal schooling were self-reported (in completed years as a proxy of adult and child socioeconomic positions, respectively) and used as continuous variables, and early nutritional supplementation with *Atole* was used as a dichotomous variable (yes/no). The original study participants were assigned to Atole or Fresco based on their village of birth.

### Statistical analysis

We evaluated the associations between the number of live births and BMI at the 1988-89, 2002-04 and 2015-17 follow-up waves using multivariable linear regression models.

#### Births through 1988-89

We ran multivariable linear regression models using the total number of live births each woman had by 1988-89 as the exposure variable and BMI in 1988-89 as the outcome. Women ≥ 15 years in 1988-89 were included in these models. We defined five models a priori with progressive adjustment for potential confounders: model 1 (unadjusted); model 2 (age-adjusted); model 3 (model 2 + own schooling); model 4 (model 3 + *Atole* exposure); and model 5 (model 4 + maternal schooling).

#### Births 1988-89 through 2002-04

We ran multivariable linear regression models using the number of live births between 1988 and 89 and 2002-04 as the exposure variable and the change in units of BMI over this same time interval as the outcome. We defined six models with progressive adjustment for potential confounders: model 1 (unadjusted); model 2 (age-adjusted); model 3 (model 2 + BMI at the beginning of period + live births in the previous period); model 4 (model 3 + own schooling); model 5 (model 4 + *Atole* exposure); and model 6 (model 5 + maternal schooling).

#### Births 2002-04 through 2015-17

 We ran multivariable linear regression models using the number of live births between 2002 and 04 and 2015-17 as the exposure variable and the change in units of BMI over this same time interval as the outcome. We assessed for confounding using the same set of models as for the period 1988-89 through 2002-04.

For each interval, we calculated the *P* value for trend using a Wald test of a continuous variable based on the median BMI change for each category of parity. We accounted for age to control for secular weight gain [3, 15] and age at first pregnancy [16], for own and maternal schooling (as a proxy of lifetime socioeconomic position) because socioeconomic position has been associated with both parity and BMI [17–20] and for nutritional supplementation consumption (*Atole)* during childhood, because there is evidence that early-life exposure to *Atole* during the first 1000 days of life increases the risk of obesity later in life [21] and also has been associated with age at first birth [22] (Fig. 1). In addition, we repeated the analysis using the cumulative number of liveborn children 1988-89 to 2015-17 as the exposure variable and the change in BMI over the same period as the outcome, in order to compare our results with other studies. As a sensitivity analysis, we carried out the same analysis using as the main outcome the change in waist circumference (centimeters) because waist circumference is a good marker of abdominal adiposity and might be a better predictor of an adverse obesity phenotype [10].

We used a complete-case analysis, assuming missingness to be completely at random. Thus, our sample represents a random sample of our study population. Though we did not conduct a formal analysis of attrition, a comparison of baseline characteristics suggest that participation was non-differential across the adult waves (1988-89, 2002-04, 2015-17).

All statistical tests were two-sided and considered statistically significant if *P* < 0.05. All analyses were conducted using Stata, version 16.0 (Stata Corp., College Station, USA).

## Results

At the 1988-89 follow-up, the mean (standard deviation, SD) age of women was 19.9 (3.2) years and about 42.1% were married or in a civil union. (Table 1). In 1988-89, among cohort members ≥15 years, women who had 2-3 children were older, and had greater BMI and waist circumference than nulliparous women (Table 2). The reproductive patterns changed over time. Between 1988 and 2002, women were in early adulthood years and had a peak of reproduction, and between 2002 and 2015 the number of reproductive events slowed (Table 2).

**Table 1:** Basic characteristics of the study population, INCAP Longitudinal Study

	Women in 1988-89 (n=498)	Women in 2002-04 (n=778)	Women in 2015-17 (n=685)
Age (y)	19.9 (3.2)	31.9 (5.3)	45.0 (4.3)
Age at menarche (y)	13.7 (1.4)	13.6 (1.4)	13.6 (1.4)
Time between menarche and first pregnancy among parous women (y)	7.4 (3.9)	7.2 (3.7)	7.3 (3.7)
Age at first birth among parous women (y)	21.1 (4.0)	20.8 (3.7)	20.7 (4.0)
BMI (kg/m^2^)	20.7 (3.4)	26.6 (4.6)	29.2 (5.1)
Waist circumference (cm)	73.6 (9.9)	91.8 (11.3)	101.7 (12.6)
Total grades completed (y)	3.6 (2.2)	3.6 (2.2)	3.4 (2.1)
Maternal Schooling (y)	1.4 (1.6)	1.5 (1.6)	1.3 (1.6)
Married/in union (%)	42.1	30.8	30.1
Atole exposure (%)	49.8	52.0	55.0

Values are mean (SD) unless otherwise specified

**Table 2:** Selected characteristics of the study population by live births in three different periods during life-course, INCAP Longitudinal Study

**Status at 1988 follow-up**					
	**Live births by the 1988-89 follow-up among women ≥15 y at the time of this survey**				
	**0 child** **(n= 422)**	**1 child** **(n= 55)**	**2 - 3 children**^a^ **(n= 21)**		
Age at the beginning of period (y)	18.9 (2.9)	22.4 (2.2)	24.4 (1.8)		
BMI (kg/m^2^)	20.4 (2.1)	21.9 (2.3)	21.0 (1.6)		
Waist circumference (cm)	72.6 (5.2)	76.1 (5.4)	73.9 (5.3)		
**Status at 2002 follow-up**					
	**Live births in the period 1988-2002**				
	**0 child** **(n=86)**	**1 child** **(n=95)**	**2 children** **(n=151)**	**3 children** **(n=173)**	**≥ 4 children** **(n=273)**
Age at the beginning of period (y)	22.8 (8.1)	19.9 (9.2)	20.6 (9.1)	22.9 (7.7)	24.7 (6.7)
Children liveborn prior to beginning of period (median, IQR)	1 (0, 3)	1 (0, 3)	0 (0, 0)	0 (0, 0)	0 (0, 0)
BMI at the beginning of period (kg/m^2^)	19.5 (3.5)	20.4 (3.5)	19.8 (3.4)	21.3 (3.6)	21.9 (3.2)
Change in BMI over period (kg/m^2^)	4.5 (3.5)	5.2 (3.4)	6.7 (4.4)	6.6 (4.4)	5.4 (4.1)
Waist circumference at the beginning of period (cm)	69.7 (8.7)	72.2 (9.7)	70.9 (9.5)	75.4 (9.7)	78.3 (9.2)
Change in waist circumference over period (cm)	14.5 (8.9)	16.8 (9.5)	20.5 (11.4)	19.2 (11.9)	15.3 (11.9)
**Status at 2015 follow-up**					
	**Live births in the period 2002-2015**				
	**0 child** **(n=346)**	**1 child** **(n=188)**	**2 children** **(n=78)**	**3 children** **(n=28)**	**≥ 4 children** **(n=45)**
Age at the beginning of period (y)	31.8 (4.3)	32.8 (4.1)	30.7 (3.6)	32.3 (4.1)	33.6 (4.4)
Children liveborn prior to beginning of period (median, IQR)	3 (2, 4)	3 (2, 4)	2 (1, 4)	2 (0, 5)	3 (2, 4)
BMI at the beginning of period (kg/m^2^)	24.8 (4.7)	26.2 (4.8)	25.9 (5.1)	27.4 (4.3)	27.4 (4.8)
Change in BMI over period (kg/m^2^)	2.3 (3.1)	1.9 (2.8)	1.9 (2.9)	2.4 (3.1)	2.5 (3.4)
Waist circumference at the beginning of period (cm)	86.8 (11.8)	89.4 (11.5)	89.5 (12.7)	92.9 (10.8)	94.2 (11.9)
Change in waist circumference over period (cm)	10.4 (6.7)	9.4 (7.2)	8.8 (7.6)	9.6 (7.8)	9.5 (8.0)

Values are mean (SD) unless otherwise specified

^a^ 2 and 3 children were merged due to low sample cases

At the time of the 2002-04 follow-up, women were 26-36 years old. Women who had four or more children within the period between 1988 and 89 and 2002-04 were older, had higher BMI and waist circumference at the beginning of the period, and were more likely to gain weight and increase their waist circumference than women that did not give birth in the same interval. Specifically, women who had 4 or more children experienced a mean (SD) BMI gain of 5.4 (4.1) kg/m^2^ and 15.3 (11.9) cm increase in waist circumference while women that did not have children in the same period had increases of 4.5 (3.5) kg/m^2^ and 14.5 (8.9) cm for BMI and waist circumference, respectively (Table 2).

There was no association of BMI change or increment in waist circumference by categories of live births in the period between 2002 and 04 and 2015-17. The mean BMI (SD) increased from 20.9 (3.4) kg/m^2^ in 1988-89 to 26.9 (4.8) kg/m^2^ in 2002-04 and 29.2 (5.1) kg/m^2^ in 2015-17, and the increment in BMI over each period increased among categories of parity (Table 2).

Parity in early adulthood was positively associated with higher increment in BMI, while there was no an association between parity and BMI at adolescence or mid adulthood (Table 3). The increment in BMI increased with the number of live births from adolescence to early adulthood, and this association was linear up to three live children, after this, weight gain reached a plateau. Compared to women who did not give birth during middle adulthood, BMI increased by 0.90 (95% CI: -0.55, 2.35), 2.39 (95% CI: 1.09, 3.70) and 2.54 (95% CI: 1.26, 3.82) for those who had one, two or three children during the same period after adjusting for confounding factors, with no differences for those who had four or more children (Table 3). After accounting for BMI at the beginning of the period and live births in the previous period (model 3) we observed that estimators strengthened, suggesting that the association between live births and change on BMI is not mediated through these variables. When we considered the whole reproductive life-course, we found that the cumulative number of liveborn children was also positively associated with the change of BMI over 1998-89 to 2015-17. Current BMI increased by 0.93 (95% CI: -0.17, 2.03) among women who had four or more children when compared with nulliparous women after accounting for confounders (Table 4).

**Table 3:** Association between number of live births and change in BMI [β(95% CI)] in three different periods during life-course, INCAP Longitudinal Study

**1988 follow-up**						
	**Live births by the 1988 follow-up (n= 498 women ≥15 y in 1988)**					**p trend**
	**0 child** **(n= 422)**	**1 child** **(n= 55)**	2 - 3 children* **(n= 21)**			
Model 1	Reference	1.58 (0.98, 2.17)	0.66 (-0.26, 1.58)			0.0001
Model 2	Reference	0.55 (-0.03, 1.13)	-0.97 (-1.87, -0.06)			0.54
Model 3	Reference	0.73 (-0.03, 1.50)	-1.42 (-3.09, 0.25)			0.88
Model 4	Reference	0.74 (-0.03, 1.52)	-1.41 (-3.09, 0.27)			0.89
Model 5	Reference	0.74 (-0.05, 1.53)	-1.41 (-3.09, 0.27)			0.94
**2002 follow-up**						
	**Live births in the period 1988-2002 (n= 778 women)**					**p trend**
	**0 child** **(n=86)**	**1 child** **(n=95)**	**2 children** **(n=151)**	**3 children** **(n=173)**	**≥ 4 children** **(n=273)**	
Model 1	Reference	0.68 (-0.75, 2.11)	2.16 (0.90, 3.41)	2.06 (0.82, 3.29)	0.86 (-0.34, 1.99)	0.94
Model 2	Reference	0.72 (-0.71, 2.15)	2.18 (0.90, 3.47)	2.35 (1.09, 3.59)	1.94 (0.71, 3.17)	0.007
Model 3	Reference	0.84 (-0.61, 2.28)	2.29 (0.99, 3.59)	2.43 (1.17, 3.70)	2.14 (0.88, 3.39)	0.002
Model 4	Reference	0.83 (-0.61, 2.28)	2.30 (0.99, 3.59)	2.42 (1.15, 3.70)	2.12 (0.85, 3.39)	0.003
Model 5	Reference	0.89 (-0.56, 2.34)	2.37 (1.07, 3.67)	2.50 (1.26, 3.82)	2.21 (0.93, 3.49)	0.002
Model 6	Reference	0.90 (-0.55, 2.35)	2.39 (1.09, 3.70)	2.54 (1.26, 3.82)	2.24 (0.95, 3.52)	0.002
**2015 follow-up**						
	**Live births in the period 2002-2015 (n=685 women)**					**p trend**
	**0 child** **(n=346)**	**1 child** **(n=188)**	**2 children** **(n=78)**	**3 children** **(n=28)**	**≥ 4 children** **(n=45)**	
Model 1	Reference	-0.28 (-1.68, 1.12)	-0.32 (-1.56, 0.93)	0.12 (-1.02, 1.28)	0.23 (-0.87, 1.32)	0.33
Model 2	Reference	0.06 (-1.30, 1.44)	-0.44 (-1.64, 0.77)	0.22 (-0.90, 1.34)	0.64 (-0.43, 1.71)	0.02
Model 3	Reference	0.12 (-1.23, 1.47)	-0.50 (-1.78, 0.78)	0.26 (-1.06, 1.57)	0.31 (-1.33, 1.96)	0.005
Model 4	Reference	0.12 (-1.24, 1.47)	-0.50 (-1.79, 0.78)	0.25 (-1.06, 1.57)	0.31 (-1.33, 1.96)	0.005
Model 5	Reference	0.16 (-1.18, 1.50)	-0.40 (-1.68, 0.87)	0.35 (-0.96, 1.66)	0.42 (-1.21, 2.06)	0.002
Model 6	Reference	0.18 (-1.16, 1.53)	-0.34 (-1.62, 0.94)	0.36 (-0.95, 1.68)	0.41 (-1.23, 2.05)	0.003

1988 follow-up:

Exposure= Live births by 1988 Outcome= BMI in 1988

Model 1= Unadjusted; Model 2= Age-adjusted; Model 3= Model 2 + schooling; Model 4= Model 3 + *atole* exposure; Model 5= Model 4 + maternal schooling

2002 follow-up:

Exposure= Live births in the period 1988-2002 Outcome= Change in units of BMI over the period 1988-2002

Model 1= Unadjusted; Model 2= Age-adjusted; Model 3= Model 2 + BMI at the beginning of period + live births in the previous period; Model 4= Model 3 + schooling; Model 5= Model 4 + *atole* exposure; Model 6= Model 5 + maternal schooling

2015 follow-up:

Exposure= Live births in the period 2002-2015 Outcome= Change in units of BMI over the period 2002-2015

Model 1= Unadjusted; Model 2= Age-adjusted; Model 3= Model 2 + BMI at the beginning of period + total live births; Model 4= Model 3 + schooling; Model 5= Model 4 + *atole* exposure; Model 6= Model 5 + maternal schooling

**Table 4:** Maternal BMI change from 1988 to 2015 [β (95% CI)] in relation to cumulative number of liveborn in the INCAP Longitudinal Study

	Cumulative number of liveborn					p trend
	**0 child** **(n=86)**	**1 child** **(n=95)**	**2 children** **(n=151)**	**3 children** **(n=173)**	**≥ 4 children** **(n=273)**	
Current BMI^a^	27.4 (5.2)	28.3 (4.7)	28.1 (5.2)	29.8 (4.8)	29.7 (5.2)	
Model 1	Reference	-0.27 (-1.68, 1.13)	-0.27 (-1.53, 0.97)	0.19 (-0.96, 1.36)	0.17 (-0.93, 1.27)	0.34
Model 2	Reference	0.06 (-1.30, 1.41)	-0.41 (-1.62, 0.80)	0.27 (-0.85, 1.39)	0.59 (-0.48, 1.66)	0.02
Model 3	Reference	0.28 (-1.11, 1.67)	-0.03 (-1.26, 1.19)	0.69 (-0.45, 1.84)	0.87 (-0.23, 1.96)	0.02
Model 4	Reference	0.30 (-1.09, 1.69)	-0.01 (-1.24, 1.21)	0.69 (-0.46, 1.83)	0.82 (-0.29, 1.92)	0.04
Model 5	Reference	0.29 (-1.09, 1.68)	0.09 (-1.12, 1.32)	0.75 (-0.39, 1.88)	0.93 (-0.17, 2.03)	0.03
Model 6	Reference	0.29 (-1.09, 1.68)	0.09 (-1.12, 1.32)	0.79 (-0.36, 1.93)	0.93 (-0.17, 2.03)	0.02

Exposure= Cumulative number of liveborn children (1988-2015) Outcome= Change in BMI over 1988-2015

Model 1= Unadjusted; Model 2= Age-adjusted; Model 3= Model 2 + BMI at 1988; Model 4= Model 3 + schooling; Model 5= Model 4 + *atole* exposure; Model 6= Model 5 + maternal schooling

^a^Data from 2015 follow-up, Mean (*SD*)

Results from the sensitivity analysis yielded similar results: parity in early adulthood was positively associated with higher increment in waist circumference, while there was no an association between parity and waist circumference at adolescence or mid adulthood (Supplementary Table 1). The increment in waist circumference (centimeters) increased with the number of live births from adolescence to early adulthood, and this association was linear up to three live children, after this, the increment in waist circumference reached a plateau. Compared to women who did not give birth during middle adulthood, waist circumference (centimeters) increased by 2.72 (95% CI: -1.09, 6.54), 6.25 (95% CI: 2.80, 9.70) and 6.99 (95% CI: 3.63, 10.37) for those who had one, two or three children during the same period after adjusting for confounding factors, with no differences for those who had four or more children (Supplementary Table 1). When we considered the whole reproductive life-course, we found that the cumulative number of liveborn children was also positively associated with the change of waist circumference over 1998-89 to 2015-17. We obtained the same estimates than obtained when modeling the association in the early adulthood (Supplementary Table 2).

## Discussion

We assessed the association between timing of live births and maternal BMI change in Guatemalan women along the life-course and we found a positive association between total number of live births during early adulthood and weight gain. Weight gain associated with live birth is probably due to excessive gestational weight gain and postpartum weight retention through repetitive reproductive cycles [23]. Our findings suggest that women who have three or more children in a short period of time during the period of peak reproduction in early adulthood (late 20s and early 30s) gain more weight compared with women who had no children in the same period. Additionally, women who have children earlier or later in their reproductive lives do not gain weight over and above natural increases associated with age.

Parity has been associated with both short- and long-term weight gain in women in other studies [2, 3, 23, 24], but the age at which women have their children has not been studied. Results from the Black Women’s Health Study in the US have shown that primiparous women with obesity and between 25 and 29 years, had BMI 1.1 kg/m^2^ greater than nulliparous women [25]. This highlights the importance of considering timing of childbirth for maternal obesity prevention. We hypothesize that the increment in BMI and waist circumference in young adult multiparous women could be due to short interpregnancy intervals that do not provide opportunity for women to return to their pre-gestational weight in the postpartum period. Weight gain and cumulative adiposity may be sensitive to the timing of pregnancy, social and contextual experiences. However, timing of pregnancy is based on multiple factors, including the couple’s age, fertility aspirations, access to health services (including family planning), childrearing support, and educational, social and economic circumstances [26, 27]. When we modeled the association between the cumulative number of live births and the change in BMI from 1988 to 2015, we found a positive association, but the estimates were attenuated compared with results obtained within the period 1988-89 through 2002-04. This highlights the importance of considering the timing of reproductive events because health outcomes may differ depending on when during the life-course reproduction occurs [28, 29], and supports the use of a life-course approach when studying reproductive (and other) health outcomes.

The increased obesity burden observed in these women could be due to the interaction between the timing of childbirths and age- and secular-trend-related biological, and contextual level factors. Women had a greater increment of BMI due to childbirths during their transition to early adulthood, the period from 1988 to the early 2000s. These women may have been more vulnerable to excessive weight gain and postpartum weight retention due to greater exposure to the obesogenic environments that promote rapid changes in dietary and physical activity patterns since 1980s in Guatemala [30–33] and may find it increasingly difficult to avoid sedentary and unhealthy patterns of behavior. The diet and physical activity patterns of the cohort participants have changed significantly over the last years, including increases in the consumption of fat and proteins [34], adoption of Western dietary patterns [35] and decreasing participation in agricultural activities [36]. This may explain the increment in BMI observed too among women who did not have any children in this interval time.

Exposure to obesogenic environments could exacerbate the biological vulnerability to excessive weight gain [37] during pregnancy and the postpartum and interpregnancy periods. Younger cohorts facing the food system transformation (impacting both supply and demand of food) and diet changes [31] may find it more difficult to adopt healthy lifestyle behaviors and avoid weight gain. This weight gain could exacerbate adverse cardio-metabolic outcomes. A significant weight gain (>5 kg) over a five-year period has been associated with cardio-metabolic risk factors, including increased body fat, dyslipidemia, and elevated blood pressure in this study population [38].

Half of Guatemalan women (50.4%) have short stature (< 150 cm) [17] and it is unknown if women with short stature are at increased risk of excessive gestational weight gain and postpartum weight retention compared with non-short women. Excessive gestational weight gain is a strong predictor of postpartum weight retention and increased weight gain in the long-term [39]. Current Guatemalan health care guidelines [40] are based on the US Institute of Medicine (IOM) recommendations for optimal gestational weight gain that account for pre-gestational BMI but do not address women of short stature [41]. The high prevalence of short stature in settings like Guatemala and other parts [42] calls for the need for revised recommendations that will help avoid excessive gestational weight gain, that contributes to the maternal obesity epidemic and adverse health consequences such as obstructed labor at childbirth, delay and short duration of breastfeeding and the intergenerational transmission of obesity [1, 43].

Our results are relevant for public health policy and clinical practice in several aspects. First, our results suggest that having a greater number of children in a short period promotes adiposity and excessive weight gain among those transitioning from adolescence to early adulthood. Therefore, interventions that promote a minimum interpregnancy interval of 24 months and provide interpregnancy care as a continuum from postpartum care are needed [44]. The postpartum and interpregnancy periods are windows of opportunity to optimize weight and health before a subsequent pregnancy [43, 44] and interventions aimed to promote adequate interpregnancy intervals have the potential to reduce the risk of adverse outcomes such as infant and child mortality and low birth weight as well as benefit maternal health. Second, our results highlight the urgent need to implement interventions aiming to prevent excessive gestational weight gain and postpartum weight retention, such as nutrition and physical activity advice and breastfeeding support [45], and to include these as core components of postpartum and interpregnancy care. After pregnancy, women should be encouraged and supported to reach their pre-gestational weight by 6 - 12 months postpartum and ultimately to achieve and maintain a healthy BMI [46]. This will help ensure that women enter a subsequent pregnancy at an optimal weight and keep or adopt healthy behaviors for the long-term. Third, improving food and built environments will help women adopt healthier lifestyles, as childrearing has been associated with changes in behavior such as increased caloric intake, consuming a less healthy diet and reduced physical activity in the postpartum period [47, 48].

Some limitations of our analysis should be considered. First, data on pre-gestational weight, post-gestational weight, and interpregnancy periods were not assessed; therefore, we were not able to differentiate between postpartum weight retention and weight gain in the subsequent period. Second, although we adjusted for age, sociodemographic characteristics and early nutritional supplementation with *Atole*, confounding by unmeasured and unknown factors such as lifestyle risk factors (e.g. diet, physical activity, breastfeeding duration) and environmental exposures cannot be ruled out. However, our study examined live births and maternal change in BMI using a life-course approach and our results build on our understanding of the importance of timing of childbirth and weight gain in women of reproductive age. In addition, using this approach has implications for the development of effective health policy that moves beyond identifying not only the type of interventions needed but also the most appropriate time across life to intervene. Third, another limitation is that meaning of education may vary for different birth cohorts [49]. Older cohorts will be overrepresented among those classified as less educated because educational attainment has improved over the years [49]. Furthermore, quality of education or the curriculum in Guatemala have changed throughout the years [36]. However, strengths of the use of education as a measure of socioeconomic position are that it is commonly collected in health survey and is the most frequently used socioeconomic indicator in obesity studies. It is also less prone to recall bias and more reliable over time than other socioeconomic position measures such as income [49]. In attempt to control this, we accounted for age. Fourth, non-differential misclassification of parity is a possibility as we did not include information about stillbirths or miscarriages. However, we evaluated data quality by checking that live births reported at each follow-up were equal or greater than live births reported in the previous wave and just 1% of women had discrepant data. Fifth, our findings are based on observational data so we cannot claim causality and there is a possibility of lack of statistical power to find associations. Six, although the INCAP Longitudinal Study has more than 50 years of follow-up and has experienced attrition, we did not find evidence to suggest that attrition affected the internal validity of our findings. Finally, our study participants were all of Ladino (Spanish-Mayan non-indigenous) heritage, which may decrease the transportability of our findings.

Future research is needed to understand the association between interpregnancy interval and maternal change in BMI (and other long-term outcomes), changes in weight trajectories based on different interpregnancy intervals (short and long), patterns of postpartum weight retention in relation to stillbirths, and to identify maternal nutrition interventions to mitigate the exacerbation of biological vulnerability of parous women to weight gain living in current obesogenic environments. In addition, futures studies should include information about miscarriages and stillbirths in order to have a complete reproductive history, and include repeated measures of adiposity in the postpartum period to enhance our understanding about the association between parity and adiposity.

Our findings suggest that childbirth can have different associations with BMI based on when it occurs during the reproductive period. There is an urgent call to health providers to increase attention to pre- and post-gestational BMI, provide advice on optimal gestational weight gain, counselling on adequate interpregnancy intervals and to help them to adopt healthy lifestyles (including breastfeeding) during antenatal, postnatal and interpregnancy periods. All these efforts could help to the prevention of obesity and associated health risks among women of reproductive age.

## Additional files

**Additional file 1.**

**Additional file 2.**

## Data Availability

The datasets generated and/or analyzed during the current study are not publicly available. There are ethical or legal restrictions on sharing a de-identified data set. We cannot anonymize the data from this cohort as all individuals come from one of four previously named villages and hence are readily re-identifiable once their demographic characteristics are known. We will not post data to a public archive, but we will make a replication data set available to researchers who agree to sign an LDUA and are covered under an IRB. Please contact the Research Center for the Prevention of Chronic Diseases (CIIPEC) at the Institute of Nutrition of Central America and Panama for requests.
