# Supplementary material for: Age at childbirth and change in BMI across the life-course: evidence from the INCAP Longitudinal Study
Source: BMC Pregnancy Childbirth. 2022 Feb 24;22:151. doi: 10.1186/s12884-022-04485-6 (PMC8876405; doi:10.1186/s12884-022-04485-6)
Supplement: Supplementary file 1 — Additional file 1. [file 12884_2022_4485_MOESM1_ESM.docx]

**Supplementary Table 1. Association between number of live births and change in waist circumference (in centimeters) [**β **(95% CI)]**

**in three different periods during life-course, INCAP Longitudinal Study**

| **1988 follow-up** | | | | | | |
| --- | --- | --- | --- | --- | --- | --- |
|  | **Live births by the 1988 follow-up (n= 498 women ≥15 y in 1988)** | | | | | **p trend** |
|  | **0 child**  **(n= 422)** | **1 child**  **(n= 55)** | **2 - 3 children***  **(n= 21)** |  |  |  |
| Model 1 | Reference | 3.60 (2.13, 5.06) | 1.38 (-0.84, 3.61) |  |  | 0.001 |
| Model 2 | Reference | 1.22 (-0.22, 2.67) | -2.48 (-4.70, -0.26) |  |  | 0.39 |
| Model 3 | Reference | 0.31 (-0.91, 1.54) | 0.39 (-2.30, 3.09) |  |  | 0.58 |
| Model 4 | Reference | 0.50 (-0.75, 1.75) | 0.57 (-2.12, 3.27) |  |  | 0.39 |
| Model 5 | Reference | 0.49 (-0.81, 1.79) | 0.50 (-2.25, 3.25) |  |  | 0.44 |
| **2002 follow-up** | | | | | | |
|  | **Live births in the period 1988-2002 (n= 778 women)** | | | | | **p trend** |
|  | **0 child**  **(n=86)** | **1 child**  **(n=95)** | **2 children**  **(n=151)** | **3 children**  **(n=173)** | **≥ 4 children**  **(n=273)** |  |
| Model 1 | Reference | 2.31 (-1.60, 6.11) | 6.14 (2.72, 9.56) | 5.16 (1.80, 8.51) | 0.71 (-2.45, 3.88) | 0.11 |
| Model 2 | Reference | 1.94 (-1.93, 5.81) | 5.63 (2.16, 9.11) | 5.77 (2.39, 9.16) | 3.81 (0.48, 7.14) | 0.09 |
| Model 3 | Reference | 2.50 (-1.31, 6.31) | 5.97 (2.53, 9.40) | 6.57 (3.23, 9.91) | 5.65 (2.30, 9.00) | 0.002 |
| Model 4 | Reference | 2.51 (-1.31, 6.33) | 5.97 (2.54, 9.40) | 6.59 (3.23, 9.94) | 5.69 (2.29, 9.07) | 0.002 |
| Model 5 | Reference | 2.67 (-1.15, 6.49) | 6.19 (2.74, 9.64) | 6.79 (3.43, 10.16) | 5.95 (2.54, 9.36) | 0.001 |
| Model 6 | Reference | 2.72 (-1.09, 6.54) | 6.25 (2.80, 9.70) | 6.99 (3.63, 10.37) | 6.09 (2.68, 9.51) | 0.001 |
| **2015 follow-up** | | | | | | |
|  | **Live births in the period 2002-2015 (n=685 women)** | | | | | **p trend** |
|  | **0 child**  **(n=346)** | **1 child**  **(n=188)** | **2 children**  **(n=78)** | **3 children**  **(n=28)** | **≥ 4 children**  **(n=45)** |  |
| Model 1 | Reference | -0.91 (-4.25, 2.43) | -1.59 (-4.58, 1.37) | -0.73 (-3.49, 2.02) | -0.89 (-3.51, 1.73) | 0.68 |
| Model 2 | Reference | -0.43 (-3.74, 2.89) | -1.80 (-4.74, 1.15) | -0.60 (-3.23, 2.13) | -0.31 (-2.91, 2.30) | 0.68 |
| Model 3 | Reference | -0.53 (-3.77, 2.71) | -1.59 (-4.60, 1.42) | 0.38 (-2.98, 3.06) | 0.56 (-2.98, 4.10) | 0.59 |
| Model 4 | Reference | -0.56 (-3.80, 2.69) | -1.61 (-4.62, 1.40) | 0.02 (-3.00, 3.04) | 0.58 (-2.95, 4.13) | 0.56 |
| Model 5 | Reference | -0.51 (-3.74, 2.72) | -1.49 (-4.49, 1.51) | 0.12 (-2.39, 3.14) | 0.67 (-2.86, 4.20) | 0.59 |
| Model 6 | Reference | -0.49 (-3.74, 2.76) | -1.28 (-4.30, 1.75) | 0.26 (-2.78, 3.29) | 0.78 (-2.77, 4.34) | 0.56 |

1988 follow-up:

Exposure= Live births by 1988 Outcome= Waist circumference in 1988

Model 1= Unadjusted; Model 2= Age adjusted; Model 3= Model 2 + BMI at 1988 + schooling; Model 4= Model 3 + *atole* exposure; Model 5= Model 4 + maternal schooling.

-------------------------------------------------------------------------------------------------------------------------------------------------------------------------------------------------

2002 follow-up:

Exposure= Live births in the period 1988-2002 Outcome= Change in centimeters of waist circumference over the period 1988-2002

Model 1= Unadjusted; Model 2= Age-adjusted; Model 3= Model 2 + BMI at the beginning of period + waist circumference at the beginning of period + live births in the previous period; Model 4= Model 3 + schooling; Model 5= Model 4 + *atole* exposure; Model 6= Model 5 + maternal schooling.

----------------------------------------------------------------------------------------------------------------------------------------------------------------------------------------------------------------------------------------------------------------------------------

2015 follow-up:

Exposure= Live births in the period 2002-2015 Outcome= Change in centimeters of waist circumference over the period 2002-2015

Model 1= Unadjusted; Model 2= Age-adjusted; Model 3= Model 2 + BMI at the beginning of period + waist circumference at the beginning of period + total live births; Model 4= Model 3 + schooling; Model 5= Model 4 + *atole* exposure; Model 6= Model 5 + maternal schooling
