# Supplementary material for: Age at childbirth and change in BMI across the life-course: evidence from the INCAP Longitudinal Study
Source: BMC Pregnancy Childbirth. 2022 Feb 24;22:151. doi: 10.1186/s12884-022-04485-6 (PMC8876405; doi:10.1186/s12884-022-04485-6)
Supplement: Supplementary file 2 — Additional file 2. [file 12884_2022_4485_MOESM2_ESM.docx]

**Supplementary Table 2. Maternal waist circumference change from 1988 to 2015 (in centimeters) [**β **(95% CI)] in relation to cumulative**

**number of liveborn in the INCAP Longitudinal Study**

|  | **Cumulative number of liveborn** | | | | | **p trend** |
| --- | --- | --- | --- | --- | --- | --- |
|  | **0 child**  **(n=86)** | **1 child**  **(n=95)** | **2 children**  **(n=151)** | **3 children**  **(n=173)** | **≥ 4 children**  **(n=273)** |  |
| Current waist circumference † | 97.3 (10.9) | 100.2 (13.1) | 98.9 (12.4) | 102.6 (11.8) | 103.1 (12.4) |  |
| Model 1 | Reference | 2.30 (-1.60, 6.21) | 6.14 (2.72, 9.56) | 5.15 (1.80, 8.50) | 0.71 (-2.45, 3.87) | 0.11 |
| Model 2 | Reference | 1.94 (-1.93, 5.82) | 5.63 (2.16, 9.11) | 5.77 (2.39, 9.15) | 3.81 (0.48, 7.14) | 0.09 |
| Model 3 | Reference | 2.49 (-1.31, 6.31) | 5.97 (2.54, 9.40) | 6.57 (3.23, 9.91) | 5.65 (2.29, 9.00) | 0.02 |
| Model 4 | Reference | 2.51 (-1.31, 6.33) | 5.97 (2.53, 9.40) | 6.59 (3.23, 9.94) | 5.69 (2.29, 9.08) | 0.02 |
| Model 5 | Reference | 2.66 (-1.15, 6.49) | 6.19 (2.75, 9.63) | 6.80 (3.43, 10.16) | 5.95 (2.54, 9.36) | 0.01 |
| Model 6 | Reference | 2.72 (-1.09, 6.54) | 6.24 (2.80, 9.69) | 6.99 (3.63, 10.36) | 6.09 (2.68, 9.51) | 0.001 |

Exposure= Cumulative number of liveborn children (1988-2015) Outcome= Change in waist circumference over 1988-2015

Model 1= Unadjusted; Model 2= Age-adjusted; Model 3= Model 2 + BMI at 1988 + waist circumference at 1988; Model 4= Model 3 + schooling; Model 5= Model 4 + *atole* exposure;

Model 6= Model 5 + maternal schooling.

†Data from 2015 follow-up, Mean (*SD*).
